# Application of HPLC with ELSD Detection for the Assessment of Azelaic Acid Impurities in Liposomal Formulation

**DOI:** 10.1155/2013/564962

**Published:** 2013-10-08

**Authors:** Stanislaw Han, Katarzyna Karlowicz-Bodalska, Dorota Szura, Lukasz Ozimek, Witold Musial

**Affiliations:** ^1^Department of Industrial Pharmacy, Wroclaw Medical University, Borowska Street 211A, 50-556 Wroclaw, Poland; ^2^Research and Development Center NOVASOME, Olsztyńska Street 5, 51-423 Wroclaw, Poland; ^3^Department of Physical Chemistry, Wroclaw Medical University, Borowska Street 211A, 50-556 Wroclaw, Poland

## Abstract

In the course of research and development of a new pharmaceutical formulation of azelaic acid in the liposomal form, we developed a rapid and accurate method for the detection of impurities using high-performance liquid chromatography. A chromatographic column from Merck (Purospher Star RP C18, 250–4 mm (5 **μ**m) was used in the assay, and the mobile phase gradient consisted of three phases: A—methanol : water (5 : 95) + 1.5% (v/v) acetic acid; B—water : methanol (5 : 95) + 1.5% (v/v) acetic acid; and C—chloroform. Detection of the impurities and the active substance was performed by an evaporative light-scattering detector. The method was validated for selectivity, system precision, method precision, limit of detection, and response rates. The proposed method can be used to detect impurities in the liposomal formulation of azelaic acid. The method enables separation of azelaic acid from the identified and unidentified impurities and from the excipients used in the drug form.

## 1. Introduction

**Figure 1:**
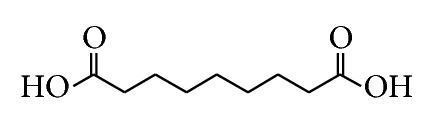
Molecule of azelaic acid.

Chemically, azelaic acid is a saturated dicarboxylic acid, also known as nonanedioic acid. Under natural conditions, azelaic acid forms on human skin from the interaction of nonanoic acid with Malassezia furfur, a microbe of the dermal bacterial flora. The azelaic acid structure is shown in Figure 1.

Azelaic acid is a relatively new therapeutic substance used in the treatment of acne. The therapeutic use of azelaic acid in the form of a 15% cream has been practiced for the past two decades. Azelaic acid has several pharmacological effects: antibacterial, keratolytic, bleaching, and metabolic [1]. The antibacterial activity is based primarily on its biocidal activity against *Propionibacterium acnes* and *Staphylococcus epidermidis*. The inhibitory effect on the 5-alpha-reductase reduces the formation of dihydrotestosterone, normally synthesised in the skin from testosterone: in effect, a reduction of the lipogenesis process in the affected tissue is observed [2, 3].

The separation and quantitative determination of the content of carboxylic acids, especially dicarboxylic acids, by chromatography have been relevant issues in analytical chemistry since the 1950s and were initially accomplished using simple systems, such as paper-based chromatography or chromatography with simple silica gel columns [4, 5]. New methods proposed for the determination of azelaic acid and other dicarboxylic acids include advanced LC, HPLC, and GC with the respective detection systems. One of the first fast GC qualitative and quantitative analyses of fatty acids was described by Metcalfe and Schmitz—it relies on the derivatisation of fatty acids to methyl esters with a solution of boron trifluoride in methanol [6].

GC combined with the derivatisation of fatty acids to methyl esters has been used to determine the amount of azelaic acid in the oil paints of old Flemish masters [7]. A similar method has been applied for evaluating the composition of wall paints [8]. Modified GC-MS has been used to study the composition of medieval and Renaissance Florentine paintings originally containing proteins and fatty acids, including azelaic acid [9]. GC-MS has also been used to determine the content of azelaic acid in samples of air from the natural environment [10, 11]. An interesting GC method for evaluating the content of azelaic acid in the natural aquatic environment was offered by Pusvaskiene et al.; they used simultaneous derivatisation and dispersive microextraction using immiscible liquids (acetone and carbon tetrachloride), whereas ethyl chloroformate was used as a reagent for derivatisation [12]. The GC method has been intensively developed due to the difficulties in providing sufficiently precise determinations [13]. The azelaic acid content in tobacco leaves has been determined by modified GC-MS using methyl derivatives and by means of solid-phase microextraction (solid-phase microextraction, SPME) [14].

Attempts to assess azelaic acid in very complex analytical matrices, for example, in biological samples of human or vegetable origin, have been made using both HPLC [15] and GC-MS methods [16]. Mansour and Ibrahiem solved the problem of simultaneous quantification of azelaic acid and benzoic acid by using isocratic, reversed-phase HPLC [17]. Ferioli et al. proposed a method for determining azelaic acid by reversed-phase HPLC (RP-HPLC) using precolumn derivatisation followed by solid-phase extraction (SPE) of a complex matrix (a pharmaceutical preparation); the derivatisation ensures detection of the active substance, which does not have a chromophore [18]. In another RP-HPLC method with a fluorescence detector, 2-bromoacetyl-6-methoxynaphthalene was used as a fluorescent marker in an aqueous micellar solution and in acetonitrile. This method enabled simultaneous determination of the azelaic acid and meglutol, that is, 3-hydroxy-3-methylglutaryl acid, in one sample [19]. In a complex mixture of carboxylic and dicarboxylic acids and amino acids, azelaic acid was assessed as a derivative of isobutyl chloroformate in GC, coupled with positive chemical ionisation MS (GC-MS-PCI) [20].

Preparation of the samples in GC methods is time consuming and requires considerable work. In addition, during the methylation of the sample, aggressive methylating reagents shorten the life of the chromatographic column and the integrity of the autosampler needle in a gas chromatograph. The mentioned disturbances cause frequent replacement of the column and needle and are associated with additional costs and time. Due to the obstacles encountered when using GC methods for dicarboxylic acid assessments, we decided to develop an alternative method to determine the limit of azelaic acid impurities in the final product—the liposomal form of azelaic acid. According to the available references, it is possible to use an evaporative light-scattering detector (ELSD) within an HPLC device to assess the fatty acids [21, 22].

In the last decade, the HPLC-ELSD method was intensively developed to analyse pharmaceutical products; the influence of the melting point and volatility of the analytes on the method efficiency was evaluated [23, 24]. Fries et al. attempted to use HPLC-ELSD to determine metabolites of 7-ethoxycoumarin from hepatocyte cell culture [25]. The use of an ELSD in the study of the lipid composition of the mixtures was introduced at the beginning of the last century [26]. Some authors compared the method of detection by refractive index (refractive index detector, RID) with the ELSD method [27]. The HPLC-ELSD method for the determination of fatty acids was suggested by Bravi and colleagues for examining vegetable oils: soybean, rice, pumpkin, and algae [21]. Fatty acids have been determined by HPLC-ELSD in raw herbal materials in the presence of saponins [22]. Wurst et al. have used this method for the determination of ethyl esters of fatty acids in the hair of alcohol-addicted persons [28]. The HPLC-ELSD method for the evaluation of serum lipids has been optimised and validated, which allows the separation and simultaneous determination of the following components in plasma: cholesterol esters, triglycerides, free cholesterol, and phosphatidylcholine [29]. The extremely complex composition of cow's milk was studied by the HPLC-ELSD method for the evaluation of phospholipids and sphingolipids that were structured in the globular form [30].

The aim of this study was to develop an analytical method that enables quick and reliable determination of the presence of azelaic acid impurities above a certain limit in new liposomal pharmaceutical formulations using HPLC with an ELSD.

## 2. Materials and Equipment

The following materials and equipment were used: Agilent Technologies 1200 HPLC; methanol (for HPLC); water (for HPLC); chloroform (for HPLC); 40% acetic acid (for HPLC); column: Merck, Purospher Star RP, C18, 250–4 mm, (5 *μ*m); reference sample of azelaic acid (AA, Fluka, serial number S38946); reference samples of impurities: pentanedioic acid (I1, Aldrich, serial number: S44628), hexanedioic acid (I2, Aldrich, serial number: 07128AJ), heptanedioic acid (I3, Aldrich, serial number: S68231), octanedioic acid (I4, Aldrich, serial number: S47580), decanedioic acid (I5, Aldrich, serial number: S69314), undecanedioic acid (I6, Aldrich, serial number: 1365985), dodecanedioic acid (I7, Aldrich, serial number: MKBB0577), tridecanedioic acid (I8, Aldrich, serial number: 03106JG), tetradecanedioic acid, and I9 (Aldrich, serial number: S29979); 0.20 *μ*m syringe filters; 2 mL syringes; and HPLC probes.

## 3. Conditions of the HPLC Analysis

In the course of the analysis, we used an Agilent Technologies 1200 high-performance liquid chromatograph with a Merck column (Purospher Star RP, C18, 250–4 mm (5 *μ*m)). Detection was performed by an ELSD. As a mobile phase, the following mixtures were used: A—methanol : water (5 : 95) + 1.5% (v/v) acetic acid, B—water : methanol (5 : 95) + 1.5% (v/v) acetic acid, C-chloroform.

Detection was performed using an ELSD. The flow of the mobile phase in the HPLC was 1.0 mL/min, the column temperature was 30°C, and the injection volume was 50 *μ*L. The temperature of the nebulisation within the ELSD was set at 40°C, whereas the flow of inert gas—nitrogen—inside the detector was set at 1.6 l/min.

## 4. Sample Preparation

The following samples were tested: the liposomal form of azelaic acid prepared without active substance (placebo, L), the liposomal form of azelaic acid with active substance (LA), a reference sample of azelaic acid (AA), and reference samples of identified impurities (I1–I9) that were prepared by dissolving them in mobile phase B (water : methanol (5 : 95) + 1.5% (v/v) acetic acid). During the evaluation of the assay method, solutions of individual impurities (I1–I9) and a mixture of standards of known impurities (I1 : I9) were used for comparison.

## 5. Results and Discussion

**Figure 2:**
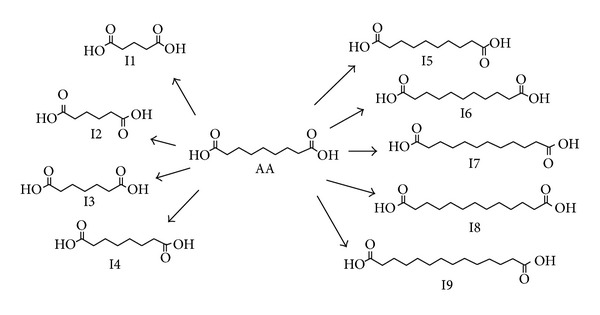
Known impurities of azelaic acid (AA)—dicarboxylic acids (I1–I9).

Impurities in the liposomal formulations of azelaic acid were determined by HPLC against a reference solution prepared by dilution of the test samples. According to the available references, the known major impurities of azelaic acid are homologous dicarboxylic acids, as shown in Figure 2.

Development of the new test method using HPLC with an ELSD included validation of the method with the following parameters: the selectivity of the method, the impact of stress factors, the precision of the system, the precision of the method, the limit of detection, and the response factors. However, validation of an assay of the limit of impurities must also include specificity, detection limit, and robustness. Validation of the method was carried out in accordance with the ICH requirements [31]. Limits were adopted on the basis of the available references and were valid for pharmaceutical products during the period of the study [32, 33].

### 5.1. Selectivity/Specificity

To determine the selectivity of the developed test method, the following evaluations of chromatograms were performed: evaluation of L, evaluation of LA, evaluation of AA, evaluation of the solutions of known impurities of azelaic acid I1 to I9, evaluation of a mixture of impurities of azelaic acid (I1 : I9), and evaluation of the mobile phase (B).

**Figure 3:**
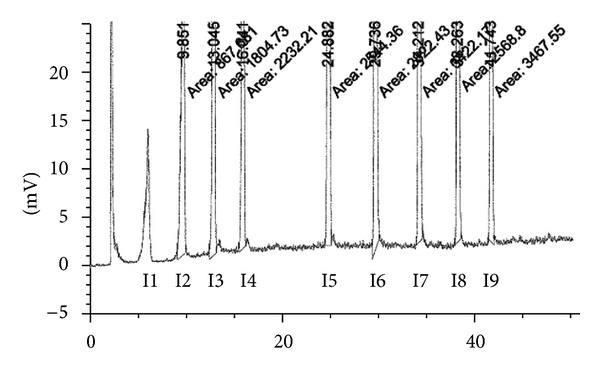
Chromatogram of the assessed known impurities, I1–I9.

During the analytical procedure, the retention times of the impurities of azelaic acid were confirmed. The expected shortest retention time, *R*_Ta_, was specified on the basis of the assessment of the glutaric acid standard solution, corresponding to the impurity I1. The longest expected retention time, *R*_Tb_, was specified according to the data from the evaluation of a standard solution of tetradecanedioic acid, I9. All the chromatograms obtained were analysed by taking into account only the peaks satisfying the condition tested: *R*_Ta_ ≤ *R*_Tx_ ≤ *R*_Tb_. An example of a chromatogram of the considered mixture of impurities is presented in Figure 3.

**Figure 4:**
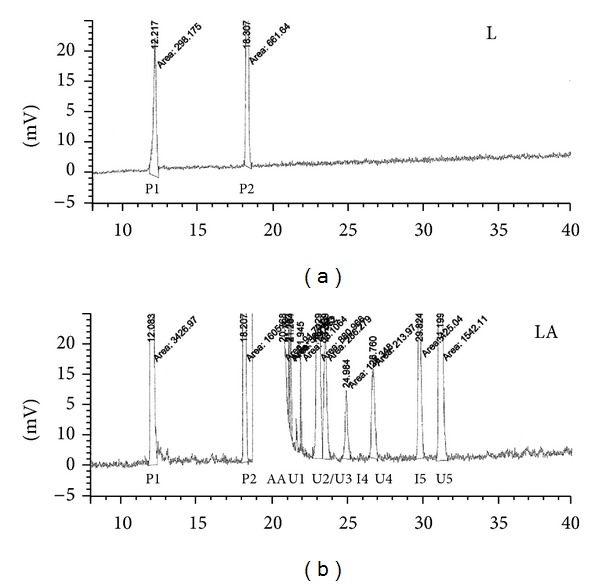
Chromatograms of HPLC-ELSD assessments: L—placebo, liposomal form of azelaic acid without the active substance; LA—liposomal form of azelaic acid; P1 and P2—impurities originating from the components of the formulation; AA—azelaic acid; I4 and I5—identified impurities; and U1–U5—unidentified impurities observed in the liposomal formulation of the azelaic acid.

As a result of the evaluation of the chromatogram of the mobile phase, it was found that there was no peak observed at the retention time characteristic of the impurities of azelaic acid. In addition, in the chromatogram of the placebo sample (L), no peaks resulting from known impurities were observed. However, two peaks were observed and were defined as P1 and P2, with retention times of *R*_*t*1_ = 12.2 min and *R*_*t*2_ = 18.3 min, respectively; the impurities P1 and P2 were identified as impurities that did not originate from the azelaic acid. The chromatogram of the sample solution—the liposomal dosage form of azelaic acid (LA)—consisted of the main peak of azelaic acid with an average retention time of 20.0 min. Two peaks were identified as similar to the peaks from the placebo sample (L) with average retention times of 12.2 min and 18.3 min, respectively. The following peaks were also observed: a peak from identified impurity I4 with an average retention time of 24.9 min, a peak from identified impurity I5 with an average retention time of 29.8 min, and the peaks of five unidentified impurities—U1, U2, U3, U4, and U5—with average retention times of 21.9 min, 23.1 min, 23.6 min, 26.8 min, and 31.2 min, respectively. Sample chromatograms of the placebo test sample (L) and the test sample (LA) are summarised in Figure 4.

Chromatograms of HPLC-ELSD assessments: L—placebo, liposomal form of azelaic acid without the active substance; LA—liposomal form of azelaic acid; P1 and P2—impurities originating from the components of the formulation; AA—azelaic acid; I4 and I5—identified impurities; and U1–U5—unidentified impurities observed in the liposomal formulation of the azelaic acid.

In the course of the subsequent analyses, we demonstrated that an unidentified impurity, U5, with an average retention time of 26.8 min occurs in the samples and that its concentration increases over time in the course of successive injections of the same solution. Because of this observation, we advise that the sample tested must be prepared immediately prior to injection.

### 5.2. Stress Tests

The following stress factors were applied to the standard solution of azelaic acid (AA) and to the sample of the liposomal formulation of azelaic acid (LA) during the 24-hour evaluation: 0.1 M HCl, 0.1 M NaOH, 3% solution of H_2_O_2_, the presence of water, and exposure to light. Furthermore, the effect of a temperature of 60°C for 1 h and the effect of sonication for 30 minutes on the sample were evaluated. On the chromatograms obtained from the analysis, it was found that the peak of the unidentified impurity U4 with an average retention time of 26.8 min appears in the standard samples of azelaic acid (AA) and in the sample (LA) in all stress conditions. Surprisingly, the peak for the unidentified impurity U5 with an average retention time of 31.2 occurs only in the LA sample. Based on this study, it can be concluded that the method meets the acceptance criteria for the determination of selectivity; the proposed test method is selective.

### 5.3. Precision of the System

The precision of the system was determined by an 8-fold analysis of the solution of nine azelaic acid impurities (I1 : I9). The results of the chromatographic system precision are shown in Table 1. The acceptance criterion was a value of RSD < 5.3%.

### 5.4. Precision of the Method

To check the precision of the analysis method, we assessed eight test samples and the reference solutions prepared according to the analytical procedure described above. In each of the samples, the location and size of the areas of the peaks of impurities were determined: two identified (I5 and I6) and three unidentified (U2, U5, and U6) with observed retention times of 23.1 min, 26.8 min, and 31.3 min. In this method, the determination of impurities of the active substance is not required to match the determination of the precision method; however, the test was performed to better understand the conditions of the test.  Table 2 summarises the results of determinations of the impurities in the test samples, along with the standard deviation and the relative standard deviation. The amount of impurities was reported as a fraction of the azelaic acid content. The results show the high precision of the test method for key impurities found in the liposomal form of azelaic acid.

### 5.5. Limit of Detection

**Figure 5:**
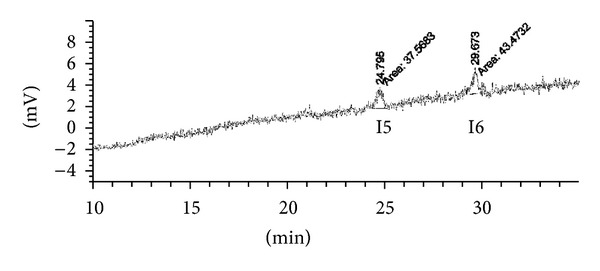
Example of the evaluation of the limits of detection, I5 and I6—identified impurities.

The limit of detection for all impurities of azelaic acid was specified as a value corresponding to the triplicate value of noise in the placebo sample (L). The chromatograms of the sample mean placebo were read from the heights of the peaks that were recorded for each impurity. Based on the signal-to-noise ratio, the theoretical concentration of analyte corresponding to the triplicate noise was calculated. Then, the solutions were prepared and analysed for concentrations of impurities. The detection limit value for each of the test substances is summarised in Table 3. The limit of detection was confirmed by analysis of the solution of the six-fold concentrations of limit of detection (LOD). An example of this analysis is shown in Figure 5.

### 5.6. Recovery Factor

Recovery factors were calculated for every identified impurity and for the azelaic acid. Subsequently, we calculated the ratios of the recovery factor (RRF) of an identified impurity (I1–I9) to the azelaic acid (AA). The values in Table 3 were calculated on the basis of a single concentration, without applying the test of linearity for different impurities. As an acceptance criterion, RRF was specified within the range of 0.8–1.2.

## 6. Conclusions

An unidentified impurity with an average retention time of 26.8 min appears in the samples and increases with time in the course of successive injections of the same solution, so the sample tested must be prepared immediately prior to injection. The method is selective, which makes it possible to identify the correct limit of azelaic acid impurities present in the liposomal form of azelaic acid intended for topical application. The precision of the system indicates that the chromatographic system is stable and is chosen properly. The precision of the method indicates that the results obtained are highly reproducible. The limits of detection confirmed experimentally for the identified impurities I1, I2, I3, I4, I5, I6, I7, I8, and I9 were 0.090 mg/mL, 0.009 mg/mL, 0.004 mg/mL, 0.005 mg/mL, 0.005 mg/mL, 0.004 mg/mL, 0.004 mg/mL, 0.002 mg/mL, and 0.003 mg/mL, respectively. This statistical method meets the statistical requirements for pharmaceutical products and provides an analytical method for determining the limit of azelaic acid impurities in the tested liposomal formulation of azelaic acid.

## Figures and Tables

**Table 1:** Results of the assessment of the precision of the chromatographic system due to the assessments of nine identified impurities of the azelaic acid.

Measurement	Peak area (A) for the assessed identified impurities: [mV × s]								
	I1	I2	I3	I4	I5	I6	I7	I8	I9
1	258.113	1203.014	1706.031	1746.713	2830.966	2752.13	4702.942	3528.136	3821.195
2	267.883	1201.028	1717.689	1736.138	2809.523	2760.488	4735.845	3499.348	3827.302
3	269.431	1207.302	1710.66	1735.395	2805.159	2751.771	4728.282	3506.829	3831.149
4	261.999	1208.041	1717.319	1757.147	2842.288	2740.871	4701.934	3511.741	3804.322
5	265.122	1193.801	1715.516	1746.554	2801.162	2787.357	4698.756	3501.03	3805.952
6	259.204	1204.02	1698.914	1756.218	2805.766	2754.597	4730.213	3490.329	3821.493
7	266.327	1193.193	1696.215	1744.038	2824.143	2751.029	4736.137	3493.413	3844.485
8	258.348	1201.998	1699.815	1743.793	2829.951	2765.384	4703.253	3532.354	3824.421

*X*	263.303	1201.550	1707.770	1745.750	2818.620	2757.953	4717.170	3507.898	3822.540
SD	4.49	5.53	8.75	7.99	15.14	13.88	16.77	15.41	13.05
RSD	1.7	0.5	0.5	0.5	0.5	0.5	0.4	0.4	0.3

I1–I9: identified impurities, *X*: average, SD: standard deviation, SRD: relative standard deviation.

**Table 2:** Results of the evaluation of the precision of the system.

Content [%]					
No. of the sample	I5 *R*_*t*_ = 24.9′	I6 *R*_*t*_ = 29.8′	U2 *R*_*t*_ = 23.1′	U5 *R*_*t*_ = 26.8′	U6 *R*_*t*_ = 31.3′
1	0.03	0.11	0.09	0.75	0.16
2	0.03	0.09	0.09	0.72	0.13
3	0.03	0.13	0.07	1.01	0.14
4	0.03	0.13	0.07	1.08	0.14
5	0.03	0.12	0.06	0.58	0.13
6	0.02	0.10	0.07	0.54	0.11
7	0.03	0.11	0.07	0.63	0.11
8	0.04	0.13	0.08	0.97	0.14

*X*	0.03	0.12	0.08	3.28	0.15
SD	0.03	0.12	0.08	1.26	0.14
RSD	0.005	0.02	0.01	1.05	0.02

I5 and I6: identified impurities; U2, U5, U6: unidentified impurities; *R*_*t*_: retention time; *X*: average; SD: standard deviation; and RSD: relative standard deviation.

**Table 3:** LODs and RRFs for the identified impurities.

Impurity	Limit of detection		RF	S (RF_I_/RF_AA_)
	[mg/mL]	[%]		
I1	0.090	0.003	4203	0.18
I2	0.009	0.005	11074	0.49
I3	0.004	0.003	19708	0.87
I4	0.005	0.005	33817	1.49
I5	0.005	0.010	27914	1.23
I6	0.004	0.012	31513	1.39
I7	0.004	0.008	40574	1.79
I8	0.002	0.004	38946	1.72
I9	0.003	0.007	46134	2.03

AA			22699	—

RF: recovery factor, RRF: ratio of the recovery factor of an impurity (RF_I_) to the recovery factor of azelaic acid (RF_AA_), I1–I9: identified impurities, and AA: azelaic acid.
